# Synthesis and numerical simulation of formamidinium-based perovskite solar cells: a predictable device performance at NIS-Egypt

**DOI:** 10.1038/s41598-023-37018-y

**Published:** 2023-06-21

**Authors:** Moamen R. A. Elsayed, Ahmed Mourtada Elseman, Alaaeldin A. Abdelmageed, H. M. Hashem, A. Hassen

**Affiliations:** 1 Faculty of Science, Department of Physics, Fayoum University, Fayoum, 63514 Egypt; 2 Photometry and Radiometry Division, Radiometry Metrology Department, National Institute of Standards, Giza, 12211 Egypt; 3 Electronic and Magnetic Materials Department, Central Metallurgical Research and Development Institute (CMRDI), Helwan, Cairo, 11421 Egypt; 4 Physics Department, Faculty of Science, Helwan University, Helwan, Cairo, 11798 Egypt

**Keywords:** Energy science and technology, Materials science, Physics

## Abstract

Formamidinium lead triiodide (δ-FAPbI_3_)-based perovskite solar cells showed remarkable potential as light harvesters for thin-film photovoltaics. Herein, the mechanochemical synthesis of *δ*-FAPbI_3_, MAPbI_3_, and mixed-cation FA_1−*x*_MA_*x*_PbI_3_ with (*x* = 0.3, 0.5, and 0.7) perovskite materials were prepared as a novel green chemistry method for scaling up production. Crystallinity, phase identification, thermal stability, optoelectronic properties, and nanoscale composition are discussed. The results demonstrated that the prepared mixed-cation samples are enhanced in the visible absorption region and are consistent with previous works. The crystal structure of *δ*-FAPbI_3_ was altered to a cubic structure due to the change in FA-cation. Moreover, the performance of $\delta$-FA-based perovskites was investigated using the Solar Cell Capacitance Simulator (SCAPS-1D) software. The validity of the device simulation was confirmed by comparing it to real-world devices. The photovoltaic characteristics and impact of absorber thickness on device performance were explained. The $\delta$-FA-based solar cell with a 50% MA-doped molar ratio shows a better performance with an efficiency of 26.22% compared to 8.43% for *δ*-FAPbI_3_. The outcome results of this work confirm the beneficial effect of mixed cations on device operation and advance our knowledge of the numerical optimization of perovskite-based solar cells.

## Introduction

Over the past few years, perovskite materials have received much attention due to their novel properties, such as high-mobility carriers, large light absorption coefficient, long carrier diffusion length, and tunable bandgap^1,2^. Because of their exceptional optical and electrical properties, halide perovskites are particularly inspiring for photovoltaic applications^3^. In 2009^4–6^, single-junction halide perovskites solar cells were reported to have a power conversion efficiency (PCE) of 3.8%, while now it has substantially increased rapidly to a remarkable certified value of 25.7%^7–9^. As a result, these materials are evolving much faster than traditional materials and might be able to take the place of crystalline silicon solar cells in global markets.

Generally, the structural formula of 3D hybrid organic–inorganic perovskites is *ABX*_3_, in which *A* is a monovalent organic/inorganic cation (e.g., CH_3_NH_3_^+^ methylammonium (MA^+^), Cesium (Cs^+1^), or HC(NH_2_)_2_^+^ formamidinium (FA^+^)), *B* is a divalent inorganic cation (usually Pb^2+^or Sn^2+^), and *X* stands for a halide (I^−^, Br^−^or Cl^−^)^10,11^. In the earlier years, MAPbI_3_ was extensively used for perovskite photovoltaic applications since the PCE of these devices exceeded 20%^12^. Pure perovskite materials, such as MAPbI_3_, have a tetragonal structure and undergo a tetragonal-to-cubic transition at a significant temperature for conventional solar cell performance^13^. However, the relatively large bandgap of MAPbI_3_ (1.55 eV up to 2.3 eV for other perovskite structures) limits its potential for further improvement. Therefore, FA^+^ is the most suitable and robust alternative for perovskite MA^+^ cation^13^. Compared to MAPbI_3_, α-FAPbI_3_ has been demonstrated to be a phase-free transition between temperature ranges. Perovskites' stability and photoelectric characteristics are governed mainly by FA^+^ and MA^+^ mixed cation perovskites. Simply stated, FA^+^ with a larger ionic radius than MA^+^ provides a sufficient and more effective replacement for MA^+^ cation in the perovskite structure. Previous tests indicated that the FA^+^ cation radii prevent development into a strong -phase and that α-FAPbI_3_ is transformed into a δ-phase^13^. When compared to MAPbI_3_, α-FAPbI_3_ has a length and long-term stability of the electron–hole dispersion. In comparison, FAPbI_3_ exhibits two crystal structures that are affected by synthesis temperatures: black-colored α-FAPbI_3_ trigonal structures and yellow-colored δ-FAPbI_3_ non-perovskite hexagonal structures.

According to the density functional theory (DFT) calculations, α-FAPbI_3_ has a smaller octahedral tilting than MAPbI_3_, resulting in a narrower bandgap (1.48 eV) for α-FAPbI_3_^14,15^. Moreover, α-FAPbI_3_ has a smaller free volume, resulting in a weaker electron-photon coupling and a longer carrier lifetime^16^. Furthermore, the calculated effective mass of α-FAPbI_3_ is lower than that of MAPbI_3_^17^, indicating superior semiconducting properties and promising perovskite material for high-performing single-junction photovoltaic applications compared to MAPbI_3_. Despite these advantages, the black-colored cubic α-FAPbI_3_ is found to be metastable at ambient temperature^13,18^. The FA^+^ replacement with MA^+^ perovskite is preferred to FAI because it has a long charge diffusion and an optimal output bandgap, which is comparable to ideal performance^13^. Therefore, as reported by Boucle *et al.*^19^, the mixed organic cation (FA^+^/MA^+^) strategy is extremely useful in stabilizing the α-FAPbI_3_, simultaneously suppressing the yellow δ-FAPbI_3_ phase, which is unsuitable for photovoltaic applications. This combination of cations quenches the spontaneous phase change from black to yellow or δ-phase, as shown later^20,21^. Due to its controlled stoichiometric ratios, a mix-cation perovskite has been suggested as an effective strategy to enhance stability and boost the PCE compared to mono-cation perovskite^13^. In particular, the incorporation of commonly known organic FA^+^/MA^+^ into the A site cation is now well established to achieve more stable and efficient photocurrent generation as compared to their pure counterparts^22^. However, to our knowledge, no studies have been conducted to fully investigate their physical, chemical, and optoelectronic properties.

In this work, two different types of δ-FA-based and a control powder sample of pristine MAPbI_3_ perovskite active layers are synthesized using a mechanochemically solvent-free solid-state reaction based on grinding method: (i) pristine δ-phase FAPbI_3_, (ii) FA_1−*x*_MA_*x*_PbI_3_, where *x* = 0.3, 0.5, and 0.7. To address the drawbacks of pure MAPbI_3_ and pure δ-FAPbI_3_, we utilized MAPbI_3_ as crystal seeds to modulate the growth of δ-FAPbI_3_ crystals and form a high-quality mixed-cation perovskite material FA_1−*x*_MA_*x*_PbI_3_ with large grain size, good crystallinity, fewer defects, and higher PCE. To know the influence of MA^+^ cation addition on δ-FAPbI_3_, crystallinity, vibrational structure, element composition, morphology, thermal stability, and optical spectra are presented. More specifically, simulations of the device’s electrical responses are performed using the Solar Cell Capacitance Simulator (SCAPS-1D) software, allowing us to discuss the influence of perovskite composition and evaluate the influences of material characteristics on the device performances of a solar cell. The influence of device parameters, such as the thickness of the absorber layer, spectral response, and external quantum efficiency, are highlighted in this study. As far as our literature review extends, this is the first report enlightening the correlation between green synthesis and SCAPS-1D simulated formamidinium organic cation-based perovskites solar devices, especially on pristine δ-phase FAPbI_3_, FA_0.7_MA_0.3_PbI_3_, FA_0.5_MA_0.5_PbI_3_, and FA_0.3_MA_0.7_PbI_3_. As a consequence, it would be meaningful for promising studies of perovskite-based optoelectronic devices.

## Experimental

### Materials

High-purity PbI_2_ (99.999% trace metals basis, yellow powder), MAI with a purity of more than (≥ 99%, anhydrous, white powder), and FAI (99.99% trace metals basis, powder) were purchased from Sigma-Aldrich Company Ltd. All the chemicals were used without further purification.

**Figure 1:**
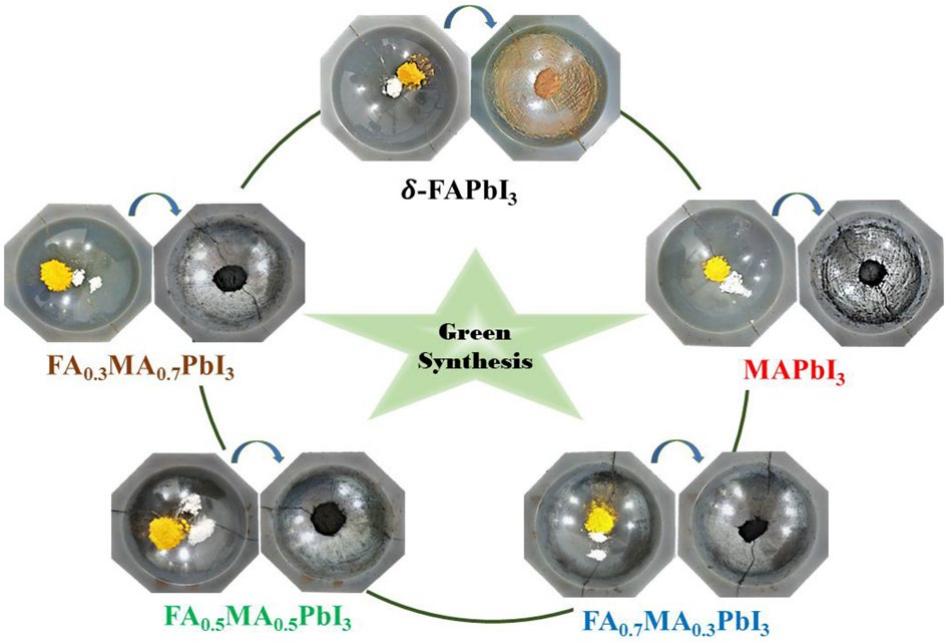
Preparation scheme of δ-FAPbI_3_, MAPbI_3_, and FA_1-*x*_MA_*x*_PbI_3_, where *x* = (0.3, 0.5, 0.7), powdered perovskite samples.

To prepare MAPbI_3_ material using the green mechanochemical synthesis approach, the high-pure precursors were weighed into a mortar (made of Zirconia to eliminate precursor contamination effectively) in stoichiometric amounts and ground together with a pestle for 50–60 min by hand at room temperature. Consequently, the black-colored MAPbI_3_ appeared by blending 2.9 g of yellow powder (PbI_2_) (1 mol) with 1.0 g of (1 mol) MAI white powder, yielding a total of 3.9 g mixture using a solvent-free solid-state approach until a magnificent structure was obtained. Correspondingly, the dark-yellow δ-phase FAPbI_3_ was prepared by adding 2.68 g of PbI_2_ (1 mol) with 1.0 g of FAI white powder (1 mol), yielding a total weight of 3.68 g mixture. As shown in Fig. 1, pure δ-FAPbI_3_ has not been synthesized successfully using this method in ambient conditions because black α-FAPbI_3_ is unstable and easily collapses into yellow δ-FAPbI_3_ structures^22^. However, a stable structure can be obtained when the proportion of MA^+^ is more than 30%. Hence, the mixed-cation perovskites with nominal compositions FA_1-*x*_MA_*x*_PbI_3_, where *x* = (0.3, 0.5, 0.7), were synthesized by 1:1:1 mol% mixtures of FAI, MAI, and PbI_2_, respectively. The mixed powder samples were made by mixing the precursors of MAI, FAI, and PbI_2_ in different ratios. Therefore, the FA_0.7_MA_0.3_PbI_3_ was made from 1 g of MAI, 2.524 g of FAI, and 9.667 g of PbI_2_. Also, the FA_0.5_MA_0.5_PbI_3_ was made from 1 g of MAI, 1.078 g of FAI, and 5.80 g of PbI_2_. The FA_0.3_MA_0.7_PbI_3_ mixture comprised 1 g of MAI, 0.46 g of FAI, and 4.14 g of PbI_2_. To avoid degradation of the samples, the ground perovskite powders were immediately transferred to quartz ampoules and evacuated, ensuring a good vacuum before the ampoules were sealed. The obtained samples are stable and homogeneous. As a result, these samples' structural and optical analyses will be relevant.

### Techniques

The crystallographic properties of the prepared samples and their phase identification were performed using an Empyrean PANalytical X-ray diffractometer (XRD) with a Cu anode x-ray source (Cu K_α1_, 1.54060 Å). Raman spectra were collected on a WITec alpha300 instrument using a laser with a 532 nm excitation wavelength at 0.1 mW laser power and an acquisition time of 5 s. The attenuated total reflectance was used to generate Fourier transform infrared (FT-IR) spectra (ATR) configuration (Platinum ATR diamond, Bruker Co.) in the range of 400 to 4000 cm^−1^. The elemental compositions and valence states of perovskite compositions were determined by using the Thermo Scientific™ K-Alpha™ XPS system. The measurements were conducted using a fully integrated, small-spot size of 400 µm with depth profiling capabilities with a base pressure of 10–9 m bar in the spectrometer and sample handler. The X-ray source is a monochromatic Al-Kα with an excitation energy of 1486.71 eV ranging from − 10 to 1350 eV, and a full-spectrum pass energy of 200 eV at a narrow spectrum of 50 eV. The microstructure of perovskite powders and elemental composition were characterized by using a field-emission scanning electron microscope (FE-SEM) and a dispersive energy X-ray (EDX) (Zeiss, Sigma 500 VP, Germany). The thermal stability of the present compositions was further confirmed by thermogravimetric and differential thermal analyses using a Shimadzu DTG-60H and Shimadzu TGA-50H in an interval from 28 to 800 °C at a heating rate of 10 °C min^−1^ under N_2_ gas with a flow rate of 30 ml min^−1^. The sample was placed in an Al_2_O_3_ crucible, and approximately 5 mg of the powder was used for each experiment. UV–vis/NIR spectrophotometer (Jasco V-570, Japan) coupled with an integrating spherical reflectance unit (ISN) in the wavelength range (200–2000) nm was used to investigate the UV–vis absorbance and diffuse reflectance spectra of several perovskite samples at ambient temperature. A 50 W xenon lamp measured the photoluminescence (PL) spectra at room temperature (Shimadzu RF-5301PC, Kyoto, Japan). All of the data was gathered at room temperature.

## Results and discussions

### Characterization

#### X-ray diffraction (XRD)

**Figure 2:**
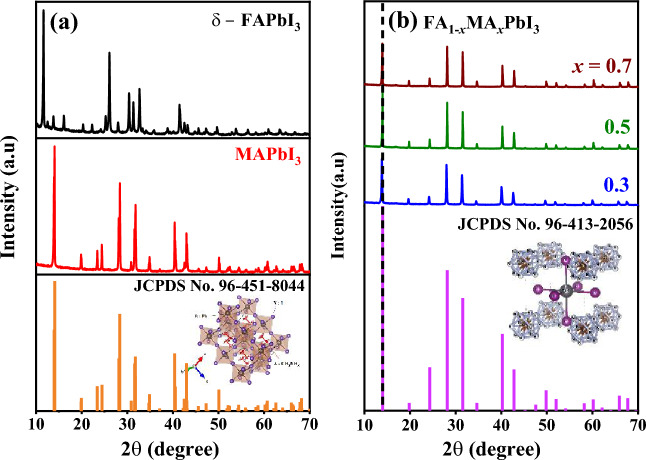
X-ray diffraction patterns of **(a)** δ-FAPbI_3_ and MAPbI_3_, matched with reference JCPDS card No. 96–451-8044, **(b)** FA_1-*x*_MA_*x*_PbI_3_, where *x* = (0.3, 0.5, 0.7), powdered perovskite samples matched with JCPDS card No. 96–413-2056.

Figure 2a,b presents the XRD patterns of pure MAPbI_3_ and FA_1−*x*_MA_*x*_PbI_3_ with (*x* = 0.3, 0.5, and 0.7). By investigating the powder XRD spectra of MAPbI_3_, the MAPbI_3_ structure is tetragonal as a symmetry group with *I4/cm* (108) space group*,* and the unit cell parameters are; *a* = *b* = 8.8718  Å, *c* = 12.6617  Å^23^. The preferred orientation along the (211) plane appeared, and the signature diffraction intensity peak of PbI_2_ in the (002) direction at 13.927˚ was observed with the highest peak. For the tetragonal structure, the reflection peaks of (211) and (310) planes were misaligned with the cubic structure, which would be useful for optimizing the cubic and tetragonal phases. The crystal structure is in good agreement with the previously published reports^24^ as well as JCPDS standard no. 96-451-8044. The XRD examination confirms high crystallinity and pure perovskite tetragonal MAPbI_3_ crystal structure. In comparison with MAPbI_3_ perovskite, $\delta$-FA-based compounds have received less attention, despite their potential. It is mainly because of the unstable nature of the pure δ-FAPbI_3_ perovskite phase and its spontaneous evolution into its yellow hexagonal polymorph (δ-phase), which has made the studies delicate. Therefore, we start this study by first inspecting the structural behavior of the yellow δ-phase of FAPbI_3_, which has a hexagonal P63/mmc symmetry^25^. In turn, the FA_1-*x*_MA_*x*_PbI_3_ structures with a molar ratio of (*x* = 0.3, 0.5, 0.7) have a crystalline cubic phase of Pm-3 m (221) space group, as displayed in Fig. 2b. The unit cells were refined to be *a* = *b* = *c* = 6.3146 Å.

To assess the effects of mixed-organic cations, the (010) plane is selected. It is observed that as the MA proportion increases, the peak positions of the XRD spectra shift slightly towards a higher angle. Because the crystal lattice size of MAPbI_3_ is smaller than that of δ-FAPbI_3_; thus, according to the Bragg equation, a higher content of MA cation FA_1−*x*_MA_*x*_PbI_3_ will result in a shift of the XRD peaks towards a higher angle^26^; as shown in Fig. 2a. Furthermore, the full width at half maximum (FWHM) narrows as the proportion of MA increases until *x* = 0.7, and then becomes wider further as the proportion of MA increases; this indicates slight differences in the crystal domain size of our samples. According to the Scherrer equation, the purer the organic cations, the larger the crystal domain. By comparing the characteristics of the XRD data of these samples, we can conclude that the FA and MA molecules are distributed uniformly within the materials. Because the molecular sizes of FA and MA are different, the strain will be induced at the boundaries between them and distributed uniformly within the crystals. The uniformly distributed strains account for the final pure cubic phase after the organic cations are mixed.

#### Raman measurements

**Figure 3:**
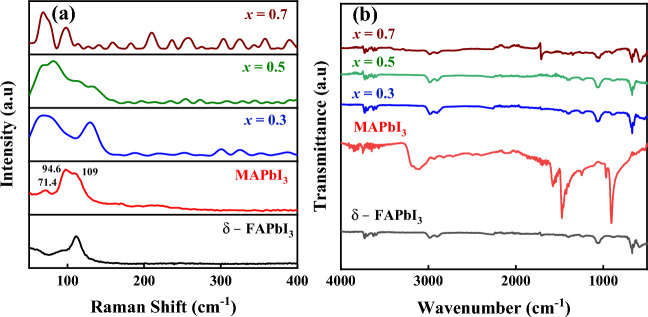
**(a)** Raman spectra, and **(b)** the attenuated total reflectance, FTIR, of δ-FAPbI_3_, MAPbI_3_, and FA_1-*x*_MA_*x*_PbI_3_, where *x* = (0.3, 0.5, 0.7), powdered perovskite samples.

Raman spectra of the investigated samples are displayed in Fig. 3a. The Raman spectra of the MAPbI_3_ powder revealed the features of rocking motion, Pb-I-Pb, bending Pb-I stretching, and the multifaceted vibration modes of the CH_3_NH_3_ cation. The peaks of the MAPbI_3_ powder are situated at 71.4, 97.5, and 109 cm^−1^, as illustrated in Fig. 3b. Notably, there are shifts in the peaks that happened for the perovskite based on FA cation, which indicated the change in the lattice of the phase transition. According to Lu et al.^27^, the Pb-I stretching forms to B_3_g symmetries, which mainly represent vibration information about the inorganic components in the material, were attributed to the peaks at 97.5 and 109 cm^−1^. The translational modes of the organic cation CH_3_NH_3_ in MAPbI_3_ are connected to the peak at 71.4 cm^−1^.

#### FT-IR analysis

FT-IR spectra of the pure MAPbI_3_ and FA_1−*x*_MA_*x*_PbI_3_ with (*x* = 0.0, 0.3, 0.5, and 0.7) perovskites were studied between 400 and 4000 cm^−1^. The structure and vibrational peaks ascribed to the substituents of perovskite materials are shown in Fig. 3b. In general, the -CH of the alkyl group displayed bending bands at 1250 and 1468 cm^−1^ for all samples, which is consistent with the symmetric bending mode of the CH_3_ and CH_2_ groups. The asymmetric and symmetric stretching modes of CH_2_ were also attributed to two significant peaks at 2920 and 2970 cm^−1^. Stretching vibration is seen in the C-N bands between 960 and 971 cm^−1^ for MAPbI_3_ and FA_1−*x*_MA_*x*_PbI_3_ with (*x* = 0.0, 0.3, 0.5, and 0.7) perovskites. The CH_3_ rocking vibration is responsible for the IR bands at 671 and 906 cm^−1^. The NH^3+^ rocking vibration was situated at 1249–1260 cm^−1^ for all samples. Furthermore, broadband has been seen at 3300–3500 cm^−1^, which has been linked to the amine groups –NH. In the infrared, the NH^3+^ bands vibrate asymmetrically at 3170 cm^−1^. The asymmetrical deformation vibration of NH^3+^ is linked to the bands at 1608 cm^−1^. The NH^3+^ scissoring vibration is linked to the bands at 1467 and 1465 cm^−1^^28,29^.

#### X-ray photoelectron spectroscopy (XPS)

**Figure 4:**
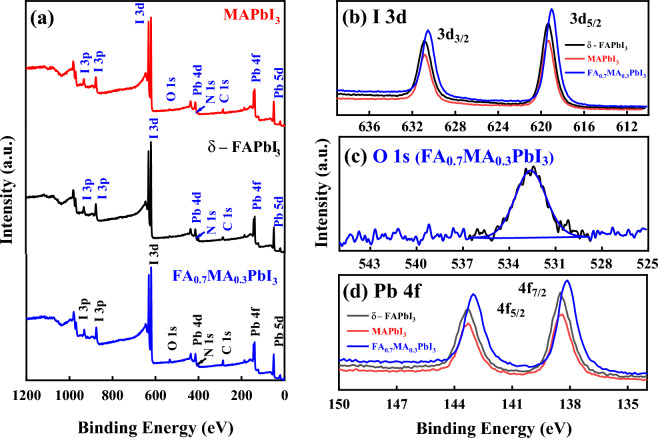
** (a)** Wide survey X-ray photoelectron spectrum (XPS), core level for **(b)** I 3d spectrum, **(c)** O 1 s spectrum, **(d)** Pb 4f. spectrum, and of δ-FAPbI_3_, MAPbI_3_, and FA_0.7_MA_0.3_PbI_3_ powdered perovskite samples.

The XPS measurements were employed on MAPbI_3_, δ-FAPbI_3_, and FA_0.7_MA_0.3_PbI_3_ perovskite samples to investigate the interaction of FA/MA organic-organic species. In addition, XPS confirmed the elemental composition after precisely small incorporation of MA material and understanding the impact of MA on the bulk effect of environmental factors on both materials and surface stability of δ-FAPbI_3_. Figure 4a depicts the broad-range XPS survey spectrum of a typical MAPbI_3_ perovskite, which reveals peaks at binding energies of 402.49 eV and 284.80 eV, respectively, matching the photoelectron peaks of N 1 s and C 1 s. Moreover, the doublet peaks of I and Pb are found with concomitant spin–orbit splitting at 619.29 eV and 138.45 eV, respectively. As a consequence, the presence of C, N, Pb, and I components in the produced powder is revealed by this broad energy spectrum, which is consistent with the earlier report^30^, illustrating the purity and complete conversion of MAPbI_3_ perovskite.

Besides, the structure is also elucidated by the elements including C, N, Pb, and I based on the spectrum of the FA_1-*x*_MA_*x*_PbI_3_ with *x* = (0.0 and 0.3)_._ According to the intensity reliance of core levels, the I 4d and Pb 5d may precisely detect the I/Pb proportions in the samples since they are the same kind of orbital. Because their binding energies are identical, the observed electrons come from the same probing depth. Unfortunately, oxygen sits on the crystal surface for the sample of *x* = 0.3 may be due to the decontamination during XPS measurements. Table 1 shows the main quantification results, including atomic concentrations of the core levels extracted from XPS survey spectra of MAPbI_3_ and FA_0.7_MA_0.3_PbI_3_. After Shirley-type background subtraction, the XPS spectra were deconvoluted with the CasaXPS program using a non-linear least-squares fitting procedure. The surface atomic percentages were determined using the manufacturer's sensitivity factors from the relevant peak regions. The narrow scan XPS spectra of the I 3d, O 1 s, and Pb 4f. regions for δ-FAPbI_3_, MAPbI_3_, and MA-doped with 0.3 are described in Fig. 4a–c. For δ-FAPbI_3_, the deconvoluted XPS spectra of the core level I 3d doublets of 3d_3/2_ and 3d_5/2_ at 619.30 and 630.75 eV, respectively, corresponding to the I_3_ charge, are illustrated in Fig. 4b. According to previous studies^31^, the lower binding component of I 3d situated at 619.30 eV is assignable to triiodide I_3_^−^ and the existence of oxidized species of iodine at the surface, which generates the I^2+^ cation and the iodate anion (IO_2_), might be indicated by the extra widening signal at 622.3 eV. The characteristic peak separation of the associated spin–orbit splitting is determined at 4.88 eV. Correspondingly, for MAPbI_3_, the same figure shows the XPS spectra of I 3d, where two peaks were observed at 619.29 and 630.76 eV, which are characteristic of 3d_5/2_ and 3d_3/2_, respectively, indicating the presence of I in the (−1) state. Accordingly, an insignificant shift of ∼0.01 eV is observed for I 3d spectrum in δ-FAPbI_3_, which might correspond to a relative effect of the local chemical environment. Additionally, for FA_0.7_MA_0.3_PbI_3_, the XPS measurement shows two peaks also for I 3d_5/2_ and 3d_3/2_ doublets spectra at 618.98 eV and 630.44 eV, respectively. Consequently, a slightly significant shift of ∼0.3 eV is observed for I 3d spectrum, which might correspond to a relatively high oxidation level. As illustrated in Fig. 4c for FA_0.7_MA_0.3_PbI_3_, the O 1 s core level was observed at 532.62 eV. The presence of O 1 s core level was found due to an oxidation level as mentioned before, or the effect of the local chemical environment.

**Table 1:** Quantification results extracted from XPS survey spectra of a perovskite δ-FAPbI_3_, MAPbI_3_, and FA_0.7_MA_0.3_PbI_3_ sample materials.

Material	Core level	Peak position (eV)	FWHM (eV)	Area	Atomic Con. (%)
δ-FAPbI_3_	I 3d	620.03	2.94	420,863.54	24
	Pb 4f.	139.17	3.07	194,733.95	12.61
	C 1 s	286.76	5.97	26,964.96	42.56
	N 1 s	401.33	2.93	22,372.83	20.83
MAPbI_3_	I 3d	619.85	2.92	321,962.04	25.73
	Pb 4f.	139.26	2.95	161,870.21	14.69
	C 1 s	286.32	4.02	22,173.65	49.05
	N 1 s	402.59	3.68	8059.96	10.53
FA_0.7_MA_0.3_PbI_3_	I 3d	619.85	2.94	471,378.36	19.34
	Pb 4f.	138.98	3.12	223,749.57	10.42
	C 1 s	286.14	3.63	39,216.34	44.52
	N 1 s	401.27	3.77	20,896.58	14.00
	O 1 s	533.36	3.58	26,321.35	11.71

Finally, for the Pb 4f. core level, in the case of δ-FAPbI_3_, there are two intense peaks located at 138.49 eV and 143.34 eV, corresponding to 4f_7/2_ and 4f_5/2_, respectively, with a distinct peak separation of 4.88 eV, indicating the presence of Pb^+2^ that could be ascribed to the Pb–I bond in perovskite^32^, as shown in Fig. 4d. At lower binding energies, the prepared material exhibits two additional peaks reflecting the presence of metallic Pb^(0)^, which is frequently detected in XPS spectra of halide perovskites^33^. Similarly, for a perovskite with MAPbI_3_ and mixed-cations of the FA_0.7_MA_0.3_PbI_3_, the Pb 4f. spectrum slightly shifts negatively to up 0.32 eV with higher binding energies.

#### Morphology and EDX analysis

**Figure 5:**
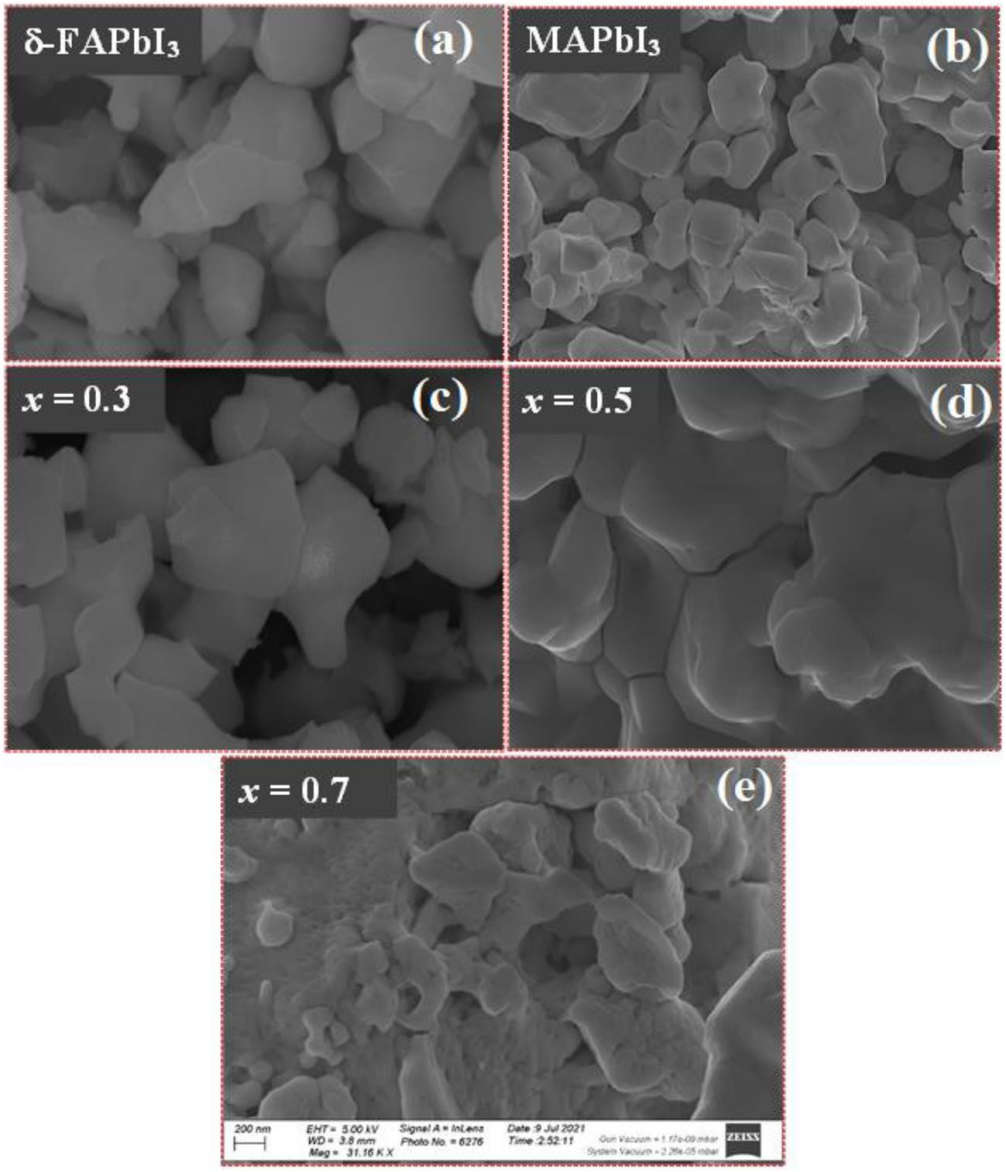
SEM images of δ-FAPbI_3_, MAPbI_3_, and FA_1-*x*_MA_*x*_PbI_3_, where *x* = (0.3, 0.5, 0.7), powdered perovskite samples. The magnification of all images is 200 nm.

**Figure 6:**
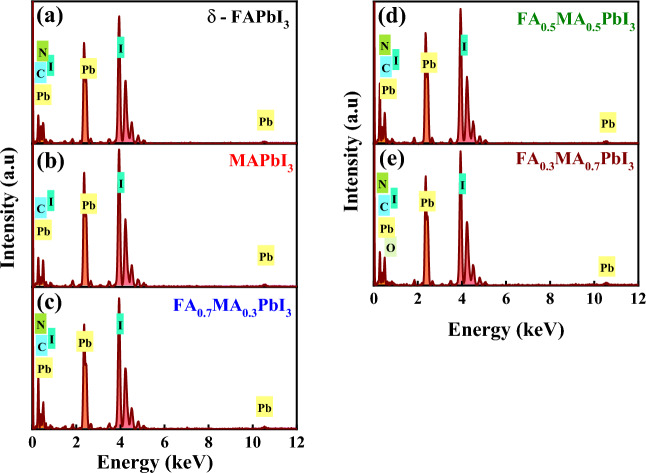
Energy dispersive X-ray (EDX) analysis of **(a)** δ-FAPbI_3_, **(b)** MAPbI_3_, and **(c-e)** FA_1-*x*_MA_*x*_PbI_3_, where *x* = (0.3, 0.5, 0.7), powder perovskite samples.

FE-SEM is used to test the morphology of the samples. The FE-SEM images of the δ-FAPbI_3_, MAPbI_3_, and FA_1−*x*_MA_*x*_PbI_3_ with (*x* = 0.3, 0.5, and 0.7) perovskite samples are displayed in Fig. 5a–e. Both pure δ-FAPbI_3_ and MAPbI_3_ showed perovskite structures with homogenous particle distribution. After adding 30% MA, tiny particles accumulated with enhancement in the grain size of the particles (see Fig. 5c). By increasing the doping of MA by 50% and 70%, the shape appears like large rocks, and some small circular rocks are scattered on it, but the small rocks seem to merge with the large ones, and that high doping can explain this has integrated with the particles of δ-FAPbI_3_ effectively, see Fig. 5d–e. SEM images demonstrate that the FA_0.5_MA_0.5_PbI_3_ has the largest crystal size, indicating that the perovskite has few grain boundary defects, which is beneficial to solar cell applications. Furthermore, some articles claim that FA_1−*x*_MA_*x*_PbI_3_ perovskites showed increased crystallinity after MA ions were incorporated into the δ-FAPbI_3_ crystal structure, which has a favorable influence on photovoltaic industries^34^. The dispersive energy X-ray (EDX) analysis was performed for more investigations, as seen in Fig. 6a–e. The analysis confirms the elemental compositions and purity of the as-prepared samples, as revealed in Table 2. The EDX results agree with the molar ratio of the elemental analysis to precisely determine the metal ratios stoichiometry of the as-prepared materials.

**Table 2:** EDX results of element compositions for pure δ-FAPbI_3_, MAPbI_3_, and FA_1−*x*_MA_*x*_PbI_3_, where *x* = (0.3, 0.5, 0.7), materials.

Composition	Wt% & At.%	Carbon	Nitrogen	Lead	Iodine	Oxygen	Total
δ-FAPbI_3_	Weight	3.33	3.20	31.58	61.90		100
	Atomic	24.18	19.92	13.31	42.59		
MAPbI_3_	Weight	3.38	1.61	32.79	62.23		
	Atomic	26.92	11.00	15.15	46.94		
*x* = 0.3	Weight	5.83	2.77	30.85	60.54		
	Atomic	37.08	15.12	11.37	36.42		
*x* = 0.5	Weight	6.37	2.58	30.90	59.87		
	Atomic	39.17	15.00	11.01	34.82		
*x* = 0.7	Weight	3.84	2.22	31.59	62.16	0.19	
	Atomic	28.24	14.01	13.46	43.26	1.03	

#### Thermogravimetric analysis

**Figure 7:**
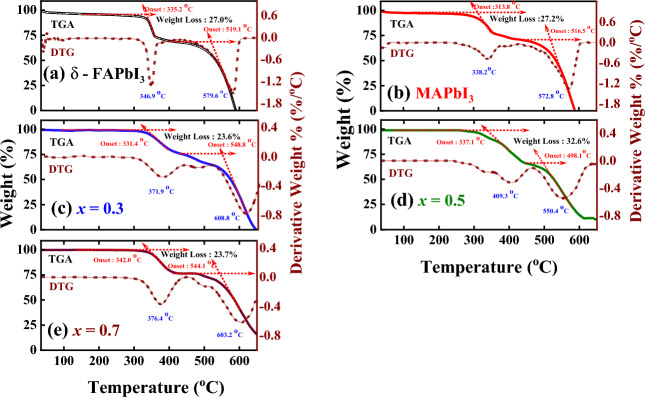
TGA (left sides) and DTG (right sides) curves of **(a)** δ-FAPbI_3_, **(b)** MAPbI_3_, and **(c-e)** FA_1-*x*_MA_*x*_PbI_3_, where *x* = (0.3, 0.5, 0.7), perovskite samples.

To investigate the thermal stability of the as-prepared perovskites, thermogravimetric analysis (TGA) was performed under nitrogen flow from room temperature to 800 °C. The TGA and derivative thermogravimetric (DTA) results showed that MAPbI_3_, δ-FAPbI_3_, and FA_1−*x*_MA_*x*_PbI_3_ perovskites decomposed before melting at the temperature range of 338.2–409.3 °C^25^, as revealed in Fig. 7a–e a small weight loss (≤ 3%) for the samples was detected may be due to the evaporation of the atmospheric moisture in the first stage. The decomposition temperature also gradually increases with the increase in MAPbI_3_ content, thereby indicating that MAPbI_3_ can increase the thermal stability of perovskites, which is good for device stability based on FA_1−*x*_MA_*x*_PbI_3_. No mass loss was detected for δ-FAPbI_3_ when the sample was heated in nitrogen until it reached 346.9 °C, see Fig. 7a. From 346.9 to 407.6 °C, the composition began to decompose through a weight loss matching FAI and PbI_2_. On the other hand, FAI begins to evaporate at a lower temperature of 278 °C for the typical precursor mixture of FAI and PbI_2_ owing to the decomposition of the unreacted free FAI to FA^+^, resulting in a weight loss equivalent to the loss of HI molecules. In the second stage, steady weight loss was detected for both compounds up to 600 °C, corresponding to the transition of PbO_2_ caused by the molecule being degraded in the air. Figure 7b depicts the thermal stability of MAPbI_3_ until 338.2 °C, after which it loses 27.2% of its initial weight at 516.5 °C. It shows that MAPbI_3_ decomposes into solid PbI_2_, along with the evolution of gaseous NH_3_ and CH_3_I^35^. Decomposition of other MAPbI_3_ perovskite samples may produce gaseous CH_3_NH_2_ and HI, leading to the loss of their structural and optoelectronic properties. Similarly, Fig. 7c–f shows that loss varies from 371.9 to 409.3 °C depending on the level of MA-doping (i.e.,* x* = (0.3, 0.5, 0.7)). Consequently, at *x* = 0.3, no mass loss until 371.9 °C before degradation to 23.6% of its initial weight at 548.8 °C, as illustrated in Fig. 7c. By increasing the doping level by 50% and 70%, the thermal stability of the samples is increased to 337.1 °C and 342.0 °C, respectively, as displayed in Fig. 7d–e. Therefore, the TGA data presented in this figure show that the addition of CH_3_NH_3_ to FA/MA mixed perovskites can actually improve their thermal stability, which is surprising considering the volatility of CH_3_NH_3_. One possible explanation for this observation is that the CH_3_NH_3_ molecules may interact with the perovskite lattice and stabilize its crystal structure. Previous studies have shown that CH_3_NH_3_ molecules can form hydrogen bonds with the halide ions in the perovskite lattice, which can enhance its stability against thermal degradation. Another possible explanation is that the CH_3_NH_3_ molecules may act as a passivation agent, reducing the number of defects and trap states in the perovskite film. This can improve the charge transport properties of the film and enhance its overall performance and stability. To further investigate these observations, additional experiments and analyses, such as XRD, FTIR and PL spectroscopy, are performed to examine the crystal structure and optoelectronic properties of the perovskite films with and without CH_3_NH_3_ addition. Overall, TGA studies reveal the thermodynamic phase purity of the synthesized δ-FAPbI_3_, and mixed FA/MA perovskites. They show high stability at ambient temperature, which is beneficial for long-term storage and commercialization. The surprising improvement in thermal stability observed in the study with CH_3_NH_3_ addition to FA/MA mixed perovskites opens up new avenues for the development of more stable and durable perovskite solar cells.

#### Optoelectronic properties

**Figure 8:**
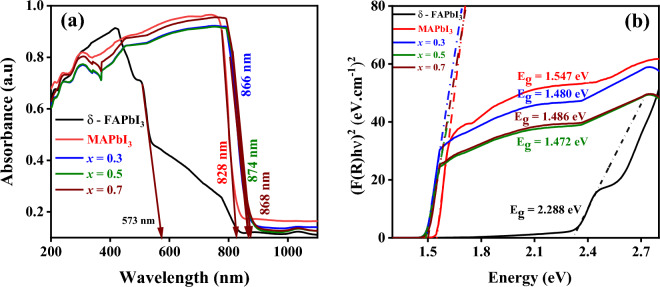
** (a)** Absorbance and **(b)** Kubelka–Munk spectrum from diffuse reflectance measurements of δ-FAPbI_3_, MAPbI_3_, and FA_1-*x*_MA_*x*_PbI_3_, where *x* = (0.3, 0.5, 0.7), perovskite samples.

UV–visible spectroscopy is a non-destructive analytical chemistry technique used for determining the quantitative composition of transition metal ions and highly conjugated organic molecules^36^. The optical properties of δ-FAPbI_3_, MAPbI_3_, and FA_1−*x*_MA_*x*_PbI_3_ with (*x* = 0.3, 0.5, and 0.7) were studied by measuring the absorbance and reflectance spectra of the synthesized powders within 200–1000 nm wavelength range. The spectra of all samples are presented in Fig. 8a,b. The absorption edge is shifted positively from 573 nm for δ-FAPbI_3_ to 866 nm for (FA-MA) mixed cation with molar ratio of 30%. After adding 50% of MA, the absorption edge still increases to 874 nm and decreases to 868 nm for 70% molar ratio. The optical absorption of δ-FAPbI_3_ has partially covered the visible region. Nevertheless, for MAPbI_3_ and FA_1−*x*_MA_*x*_PbI_3_, the optical absorption covers the entire visible as well as near IR regions. Consequently, the MA-doped samples have higher absorption in the visible range compared to the typical MAPbI_3_. In addition, the optical bandgap energy (*E*_g_) of the as-prepared samples was estimated from diffuse reflectance UV–vis data and the Kubelka–Munk equation as shown in the following formulae^24^:

1 $$F\left(R\right)=\frac{\alpha }{S}=\frac{{\left(1-R\right)}^{2}}{2R}$$

2 $${(F\left(R\right)h\vartheta )}^{n}=A\left(h\vartheta -{E}_{g}\right)$$

where *R, α,* and *S* denote reflected light, absorption, and scattering coefficients, respectively. The value *n* equals 0.5 or 2 depending on direct or indirect bandgaps. Figure 8b shows the *E*_g_ values of 2.288 eV and 1.547 eV for δ-FAPbI_3_ and MAPbI_3_ samples, respectively, which is in good consistency with previous reports^37^. Besides, FA_1−*x*_MA_*x*_PbI_3_ with *x* = (0.3, 0.5, 0.7), the values of *E*_g_ are 1.480 eV,1.472 eV, and 1.486 eV, respectively. Therefore, we observed that the FA_1−*x*_MA_*x*_PbI_3_ powders have the highest absorbance intensity and the broadest absorbance spectrum, followed by the MAPbI_3_ and δ-FAPbI_3_ perovskites, which have the narrowest absorbance spectrum. In addition, as illustrated in Fig. 8b, it is possible to state that the energy bandgap for MA-doped with *x* = 0.5 is smaller than that of the others, implying its suitable applications for solar cells.

#### Photoluminescence analysis

**Figure 9:**
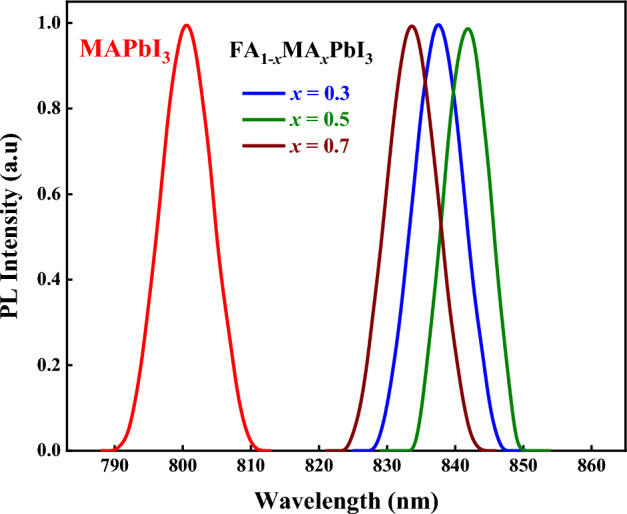
Photoluminescence results of MAPbI_3_ and FA_1-*x*_MA_*x*_PbI_3_, where *x* = (0.3, 0.5, 0.7), perovskite samples.

Photoluminescence (PL) measurements were conducted at room temperature for MAPbI_3_ and FA_1−*x*_MA_*x*_PbI_3_, as shown in Fig. 9. The measurement excitation source is a diode laser with a power of 10 mW that operates at 660 nm. For pure MAPbI_3_, single-peak emission appears at ~ 800 nm, whereas for the mixed-organic cation perovskites, single emission peak appears at the wavelength range from 834 to 842 nm positions. Stokes shifts are very small for all samples; this indicates the recombination of free excitons^38^. The FWHM of PL spectra for pure and mixed-organic cation perovskites is narrow (~ 7 nm) due to the exciton-phonon interaction. The PL peak positions for mixed organic cation perovskite materials shift positively as the MA proportion increases. This deviation may indicate a significant difference in the exciton binding energy as the organic cation proportion changes.

### Numerical simulation

Several simulation models were used in PVs technology, including AMPS (Analysis of Microelectronic and Photonic Structure), MULTIPHYSICS, COMSOL, SCAPS-1D (One-Dimensional Solar Cell Capacitance Simulator), GPVDM, TiberCAD and SILVACO, Matlab, and wxAMPS^39^. SCAPS-1D is widely regarded as the most realistic modeling and simulation technique for various types and structures of metal halide perovskite solar cells. One of the drawbacks of SCAPS-1D that we have encountered is the inability to generate the dark J-V curves and the failure to account for reflection at interfaces, which causes an underestimation of the short-circuit current density (J_sc_)^40^. In this work, the SCAPS-1D simulator was also used to predict and optimize the optoelectronic performance of our synthesized materials, as well as to investigate the impact of the absorber thickness on the device performance^41^. We employed the values of measured energy band gaps obtained from the above-mentioned mechanochemical synthesis approach as input parameters in SCAPS to simulate the performance of perovskite devices. It seems to be a significant gap between the energy band gap of perovskite powders and thin-film devices. Hence, as stated later, we assume three interface layers to analog the real devices by increasing the defect densities as input variables.

**Figure 10:**
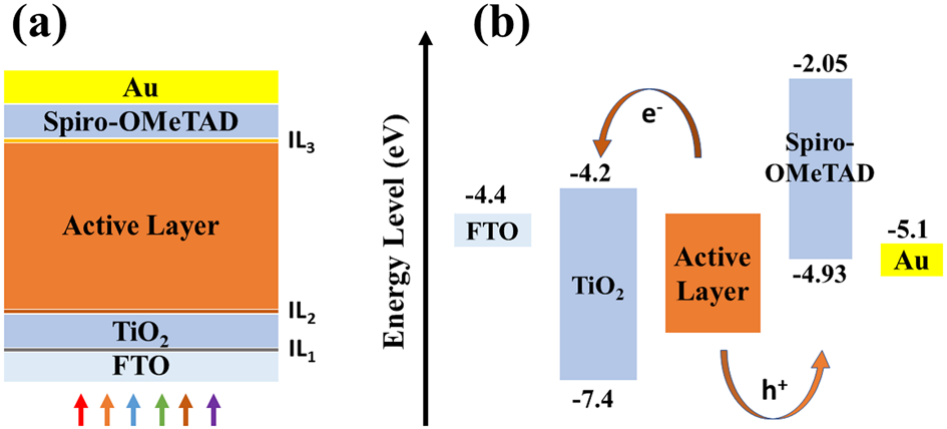
** (a)** One-dimensional n-i-p planar device configuration of the simulated PSC and **(b)** Energy band diagram of the device.

One-dimensional planar n–i–p perovskite devices (FTO/IL_1_/TiO_2_/IL_2_/Perovskites/IL_3_/Spiro-OMeTAD/Au) were simulated using SCAPS-1D software. The device configuration and energy band diagram are illustrated in Fig. 10a,b. Three very thin interface layers (IL_1_, IL_2_, and IL_3_) have been introduced in the model to mimic the defective interface between the absorber layer and transport layers. FAPbI_3_, MAPbI_3,_ and the three different types of FA-based perovskite absorber layers were used, namely FA_0.7_MA_0.3_PbI_3_, FA_0.5_MA_0.5_PbI_3_, FA_0.3_MA_0.7_PbI_3_. For simplicity, the five devices are referred to D_s_ (A), D_s_ (B), D_s_ (C), D_s_ (D), and D_s_ (E), respectively, for the rest of this study. In this device structure, MAPbI_3_ or FA-based perovskites light-harvesting material is sandwiched between two charge transport layers such as Spiro-OMeTAD as hole transport layer (HTL) and TiO_2_ as electron transport layer (ETL)^42^. However, Fluorine-doped Tin Oxide (FTO) is used as the front contact and the gold (Au) thin layer as the back metal contact.

The simulation is performed at 300-k under one sun (AM1.5G illumination and 1000 W/m^2^) irradiance in SCAPS-1D (ver.3.3.09) simulation software developed by the University of Gent, Belgium^43,44^. The numerical internal calculations are mainly derived from three coupled differential equations, namely Poisson’s Eq. (1) and continuity equation for holes (2) and electrons (3)^45–47^, as shown below.

3 $$\frac{d}{dx}\left(-\varepsilon (x)\frac{d\psi }{dx}\right)=q\left[p\left(x\right)-n\left(x\right)+{N}_{d}^{+}\left(x\right)-{N}_{a}^{-}\left(x\right)+{p}_{t}\left(x\right)-{n}_{t}(x)\right]$$

4 $$\frac{d{p}_{n}}{dt}={G}_{p}-\frac{{p}_{n}-{p}_{n0}}{{\tau }_{p}}-{p}_{n}{\mu }_{p}\frac{d\xi }{dx}-{\mu }_{p}\xi \frac{d{p}_{n}}{dx}+{D}_{p}\frac{{d}^{2}{p}_{n}}{d{x}^{2}}$$

5 $$\frac{d{n}_{p}}{dt}={G}_{n}-\frac{{n}_{p}-{n}_{p0}}{{\tau }_{n}}+{n}_{p}{\mu }_{n}\frac{d\xi }{dx}+{\mu }_{n}\xi \frac{d{n}_{p}}{dx}+{D}_{n}\frac{{d}^{2}{n}_{p}}{d{x}^{2}}$$

where $\varepsilon$ is permittivity, $q$ is the electron charge, $G$ is generation rate, $D$ is diffusion coefficient, $\psi$ is electrostatic potential, $\xi$ is the electric field, $p\left(x\right), n\left(x\right), {p}_{t}\left(x\right), {p}_{n}\left(x\right)$ are free holes, free electrons, trapped holes, and trapped electrons, respectively. ${N}_{d}^{+}$ stands for ionized donor-like doping concentration and ${N}_{a}^{-}$ refers to ionized acceptor-like doping concentration. $x$ is the direction along the thickness.

The optical bandgap energy of the light-harvesting materials (active layers) is obtained from our experimental absorption spectra measurements, which strongly influence the photovoltaic performance of these cells, according to Shockley and Queisser^48^. Basic parameters used to perform simulation work gathered from various experimental and theoretical published works are summarized in Table 3^49–55^, to firstly validate and verify our software^50,56^. The values for the defects in active layers used to simulate the work, as well as the considered interface defect density in the device simulation, is presented in Table 4. The absorption coefficient spectra ($\alpha$) of all layers is calculated in SCAPS-1D via $\alpha ={{A}_{\alpha }(E-{E}_{g})}^{1/2}$ and the prefactors ${A}_{\alpha }$ was assumed to be 10^5^. This assumption was used as in many previous studies on perovskite-based solar cells^50^. In this simulation model, ideal perovskite devices are meticulously optimized without considering *R*_*series*_ and *R*_*shunt*_ resistances. This impractical step is a key factor in determining the maximum limit of device performance achieving highly stable photovoltaic performance parameters when considering optimal operation^51^. To validate and verify our simulated model, the recently reported experimental works for the similar planar n-i-p structure (FTO/TiO_2_/FAPbI_3_ or MAPbI_3_/Spiro-OMeTAD/Au) obtained by Michael Grätzel *et. al*.^57^ and NREL cell efficiency data^7^, respectively, are compared to those obtained by SCAPS-1D. Table 5 shows the validated photovoltaic characteristics of solar cell devices reported in the literature and our simulated SCAPS. The thickness of the FTO, ETL, absorber, and HTL layers are chosen to be 100 nm, 50 nm, 800 nm, and 50 nm, respectively. The comparative current–voltage (J–V) characteristic data are shown in Table 4 with the four performance indicators, open circuit voltage (V_OC_), short circuit current density (J_SC_), fill factor (FF), and efficiency ($\eta$). Comparing the data, it is confirmed that our benchmarked model achieves convincing similarity with at least 99.001% consistency. As a result, it can affirm the feasibility of the device configuration and material parameters used in our modeling work.

**Table 3:** Electrical properties of the materials used in our simulated structures^49–55^.

Material Property	FTO	TiO_2_ (ETL)	D_*s*_ (A)	D_*s*_ (B)	D_*s*_ (C)	D_*s*_ (D)	D_*s*_ (E)	Spiro_OMeTAD (HTL) #
Thickness, $t$ (nm)	100.0	50.0	800.0	800.0	800.0	800.0	800.0	50.0
Optical bandgap Energy, ${E}_{g}$ (eV)	3.5	3.2	2.288*	1.547*	1.480*	1.472*	1.486*	3.17
Electron affinity, $\chi$ (eV)	4.0	4.0	4.0	3.9	4.0	4.0	4.0	2.2
Relative Dielectric permittivity,${\varepsilon }_{r}$	9.0	10.0	6.6	10.0	6.6	6.6	6.6	3.0
Effective cond. band density of states, ${N}_{c}$(cm^−3^)	2.2 × 10^18^	2.2 × 10^18^	1.2 × 10^19^	2.2 × 10^18^	1.2 × 10^19^	1.2 × 10^19^	1.2 × 10^19^	2.2 × 10^18^
Effective valence band density of states, ${N}_{v}$ (cm^−3^)	1.8 × 10^19^	1.8 × 10^19^	2.9 × 10^18^	1.8 × 10^19^	2.9 × 10^18^	2.9 × 10^18^	2.9 × 10^18^	1.8 × 10^19^
Electron thermal velocity, ${\nu }_{th(n)}$ (cm s^−1^)	1.0 × 10^7^	1.0 × 10^7^	1.0 × 10^7^	1.0 × 10^7^	1.0 × 10^7^	1.0 × 10^7^	1.0 × 10^7^	1.0 × 10^7^
Hole thermal velocity, ${\nu }_{th(p)}$ (cm s^−1^)	1.0 × 10^7^	1.0 × 10^7^	1.0 × 10^7^	1.0 × 10^7^	1.0 × 10^7^	1.0 × 10^7^	1.0 × 10^7^	1.0 × 10^7^
Electron mobility, ${\mu }_{n}$ (cm^2^ V^−1^ s^−1^)	20.0	20.0	27.0	2.0	27.0	27.0	27.0	2.0 × 10^–4^
Hole mobility, ${\mu }_{p}$ (cm^2^ V^−1^ s^−1^)	10.0	10.0	27.0	1.0	27.0	27.0	27.0	2.0 × 10^–4^
Shallow uniform donor density, ${N}_{D}$ (cm^−3^)	2.1 × 10^19^	5.0 × 10^19^	1.3 × 10^16^	5.21 × 10^9^	1.3 × 10^16^	1.3 × 10^16^	1.3 × 10^16^	0.0
Shallow uniform acceptor density, ${N}_{A}$ (cm^−3^)	0.0	0.0	1.4 × 10^16^	5.21 × 10^9^	1.4 × 10^16^	1.4 × 10^16^	1.4 × 10^16^	2.0 × 10^18^
Total defect density, ${N}_{t}$ (cm^−3^)	1.0 × 10^15^	1.0 × 10^15^	4.0 × 10^13^	2.5e^+13^	4.0 × 10^13^	4.0 × 10^13^	4.0 × 10^13^	1.0 × 10^15^
Defect type	Neutral	Neutral	Neutral	Neutral	Neutral	Neutral	Neutral	Neutral
Characteristic energy, (eV)	0.1	0.1	0.1	0.1	0.1	0.1	0.1	0.1
Capture cross-section electrons, (cm^2^)	1.0 × 10^–15^	1.0 × 10^–15^	1.0 × 10^–15^	1.0 × 10^–15^	1.0 × 10^–15^	1.0 × 10^–15^	1.0 × 10^–15^	1.0 × 10^–15^
Capture cross-section holes, (cm^2^)	1.0 × 10^–15^	1.0 × 10^–15^	1.0 × 10^–15^	1.0 × 10^–15^	1.0 × 10^–15^	1.0 × 10^–15^	1.0 × 10^–15^	1.0 × 10^–15^
Energetic distribution	Single	Single	Gaussian	Gaussian	Gaussian	Gaussian	Gaussian	Single
Energy level concerning the reference, (eV)	0.6	0.6	0.6	0.6	0.6	0.6	0.6	0.6

* Indicates this current work & D_*s*_ (A): FAPbI_3_, D_*s*_ (B): MAPbI_3_, D_*s*_ (C): FA_0.7_MA_0.3_PbI_3_, D_*s*_ (D): FA_0.5_MA_0.5_PbI_3_, D_*s*_ (E): FA_0.3_MA_0.7_PbI_3._ # Taken from Refs.^58,59^.

**Table 4:** Defect density values inside the active layer and at the interface of the device.

Parameters/Interfaces	Spiro-OMeTAD/Perovskite	Perovskite /TiO_2_	TiO_2_/FTO	Perovskite
Defect type	Neutral	Neutral	Neutral	Neutral
Capture cross-section electrons (cm^2^)	10^–20^	10^–20^	10^–20^	10^–15^
Capture cross-section holes (cm^2^)	10^–20^	10^–20^	10^–20^	10^–15^
Energetic Distribution	Single	Single	Single	Gaussian
Energy concerning the reference (eV)	0.6	0.6	0.6	0.6
Characteristic Energy (eV)	NA	NA	NA	0.1
Total Density (cm^-3^)	10^13^	10^13^	10^13^	4 × 10^13^

**Table 5:** Photovoltaic characteristics validation results of fabricated, reported from literature, and SCAPS simulated solar cell devices for FAPbI_3_ and MAPbI_3_, respectively, measured under standard AM 1.5G illumination at 100 mW cm^-2^ irradiance.

Device Architecture	*V*_*OC*_ (*V*)	*J*_*SC*_ (mA cm^−2^)	*FF* (%)	*PCE* (*%*)	Reference No
FAPbI_3_ (Simulated)	1.183	26.25	82.37	25.56	This work
FAPbI_3_ (Experimental)	1.189	26.35	81.70	25.59	^57^
MAPbI_3_ (Simulated)	1.240	25.30	81.22	25.49	This work
MAPbI_3_ (Experimental)	1.189	25.68	83.00	25.50	^6^

#### PSC Performance Analysis and Impact of the Absorber Thickness

**Figure 11:**
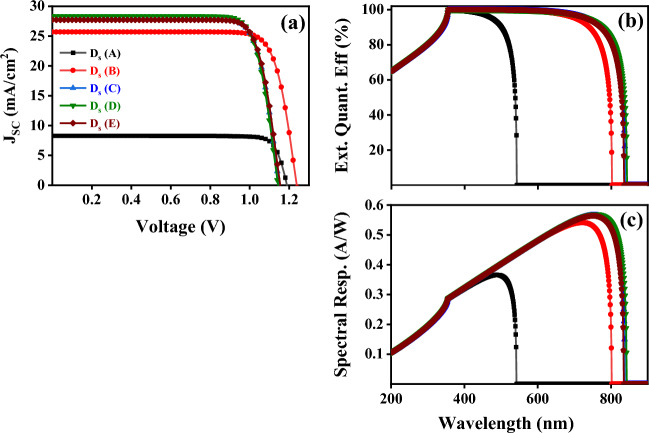
** (a)** (J–V) simulated curves, **(b)** variation of external quantum efficiency (EQE) with wavelength, and **(c)** calculated spectral power responsivity for D_s_ (A), D_s_ (B), D_s_ (C), D_s_ (D) and D_s_ (E) solar cell devices using SCAPS-1D software.

In this simulation study, the current density–voltage (J-V) curves of the n–i–p planar perovskite solar cells based on FA_1−*x*_MA_*x*_PbI_3_ absorber layers with different MAPbI_3_ concentrations are shown in Fig. 11a. The detailed obtained photovoltaic parameters of the solar cells are summarized in Table 6. The device based on the pristine FAPbI_3_ perovskite layer, D_s_ (A), shows an open-circuit voltage (V_OC_) of 1.188 V, a short-circuit current (J_SC_) of 8.28 mA⋅cm^−2^, a fill factor (FF) of 85.67% and a power conversion efficiency (PCE) of 8.43%. For the device based on the pristine MAPbI_3_ perovskite layer, D_s_ (B), achieved a V_OC_ of 1.238 V, J_SC_ of 25.70 mA cm^−2^, FF of 81.15%, and PCE of 25.83%. On the other hand, the formamidinium-based perovskite solar cell with a molar ratio of 30%, D_s_ (C), has achieved a PCE of 25.99%, a V_OC_ of 1.150 V, J_SC_ of 27.87 mA cm^−2^, FF of 81.09%. Moreover, by increasing the molar ratio to 50%, the D_s_ (D) device has reached a higher PCE of 26.22% with a V_OC_ of 1.144 V, J_SC_ of 28.34 mA cm^−2^, and FF of 80.91%. After increasing the molar ratio up to 70%, D_s_ (E), it was observed a slight decrease in PCE with a value of 25.98% and a V_OC_ of 1.154 V, J_SC_ of 27.71 mA cm^−2^, FF of 81.20%. The corresponding external quantum efficiency (EQE) spectrum of these devices in the wavelength range from 200 to 900 nm was obtained over the AM 1.5 photon flux in Fig. 11b.

**Table 6:** Final simulated performance results of D_s_ (A), D_s_ (B), D_s_ (C), D_s_ (D), and D_s_ (E) solar cell devices after feeding our experimentally obtained bandgap energies.

Simulated Device	V_OC_ (V)	V_MPP_ (V)	J_SC_ (mA cm^−2^)	J_MPP_ (mA cm^-2^)	FF (%)	PCE (%)
D_s_ (A)	1.189	1.06	8.280	7.94	85.67	8.43
D_s_ (B)	1.238	1.05	25.70	24.61	81.15	25.83
D_s_ (C)	1.150	0.97	27.87	26.85	81.09	25.99
D_s_ (D)	1.144	0.96	28.34	27.28	80.91	26.22
D_s_ (E)	1.154	0.97	27.71	26.71	81.20	25.98

Figure 11c shows the spectral power responsivity in A/W for all simulated devices^60^. Due to the use of two wide and narrow bandgap absorber layers, the optical absorption edge of the device shifts from 540 nm to more than 840 nm. It can be seen that a preferable quantum efficiency in the visible wavelength range was obtained for the perovskite structures. The absorption edge of FA-based devices is about 300 nm higher than FAPbI_3_ structure which is due to the light absorption of the latter that has a lower bandgap of 2.288 eV compared to ~ 1.5 eV. In conclusion, the champion perovskite solar cell based on FA_0.5_MA_0.5_PbI_3_ absorber layer with a thickness of 800 nm has achieved the highest PCE of 26.22% compared to other devices^61,62^. Based on it, this device has a good application prospect in the solar cell field.

**Figure 12:**
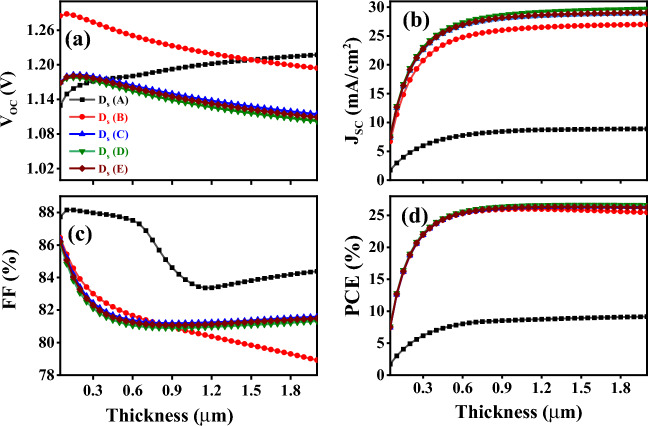
Variation of thickness for absorber layers on photovoltaic parameters: **(a)** V_OC_, **(b)** J_SC_, **(c)** FF, and **(d)** PCE.

The thickness of the absorber layer has the most crucial parameter and has a remarkable influence on the solar cell performance. Hence, in this modeling, we focus only on the impact of variation of thickness on the photovoltaic performance (i.e., V_OC_, J_SC_, FF, and PCE) of the above-mentioned devices. Keeping all electrical parameters constant as listed in Table 3, the thickness of all five simulated devices is varying from 0.05 to 2.0 $\mathrm{\mu m}$. The results of this variation in thickness are illustrated in Fig. 12a–d. As shown in Fig. 12a, the simulation results illustrate that with absorber thickness increasing, open-circuit voltage (V_OC_) increases up to 0.2 $\mathrm{\mu m}$. Beyond this thickness, V_OC_ starts to decrease slightly, except for Ds (A) device is continuously increasing. In the V_OC_ increase stage, the hole-electron recombination is lower with thinner absorber layers, keeping the dark saturation current (I_0_) at a low level. As a consequence, different excess carrier concentrations are a benefit to generate a higher light-generated current (I_L_) and promote the rising of V_OC_. However, in the V_OC_ decrease stage, the thicker absorber tends to raise I_0_ to a higher level and provides more opportunity for carrier recombination, resulting in a sharp decrease in V_OC_. On the other hand, Fig. 12b indicates that with absorber thickness increasing, short-current density (J_SC_) is continuously increasing. In the thin perovskite layer, the charge carrier diffusion length is greater than the thickness, and most of the extra carriers can reach both electrodes and generate power. The increase in thickness causes more light absorption and more extra carrier concentration, which brings J_SC_ values to upraise. Therefore, by increasing the photogenerated carriers, the value of J_SC_ increases. Moreover, for FA-based devices, the fill factor continuously drops from 86.45 to 81.07% with the thickness varying from 0.5 to 0.9 $\mathrm{\mu m}$, see Fig. 12c.

The MAPbI_3_-based device, D_S_ (B), fill factor has quite a similar behavior except that it was continuously decreased with increasing thickness. Moreover, D_s_ (A) device has a reverse bell-like-shape behavior, FF% sharply decreased until it reaches 83.37% at a thickness of 1.15 $\mu m$, then, exponentially increasing with the increasing of the layer thickness. The fill Factor is considered as an ability to deliver available power to a load generated by a cell, in other words, internal power depletion. In thicker absorbers, the internal power depletion enhances and causes a reduction of the fill factor. Figure 12d shows that the PCE% characteristic of all structures follows the same trend and is particularly identical from 0.05 to 1.2 μm of the absorber layer thickness except for device D_S_ (A). As observed in Fig. 12d, D_S_ (A) device has a very low efficiency compared to other devices due to its bandgap energy being very high (*E*_*g*_ = 2.288 eV). Besides, by increasing the absorber thickness in the solar cell, the PCE increases until it reaches nearly 800 nm. After that, it slightly impacts the PV parameters in the (0.8–2.0) μm regime.

## Conclusions

In this work, we demonstrated a novel and promising green chemical strategy for producing $\delta$-FAPbI_3_, MAPbI_3,_ and $\delta$-FA-based perovskite materials using a mechanochemical synthesis technique. The results showed that the obtained mixed-cation FA_1−*x*_MA_*x*_PbI_3_ with (*x* = 0.3, 0.5, and 0.7) perovskites using this strategy have achieved excellent crystallinity and purity as evidenced by different characterization techniques such as XRD, SEM–EDX, FTIR, XPS, and Raman spectroscopy. TGA measurements showed the thermal stability of the prepared samples within the range from 346.9 to 409.3 °C, depending on the rate of doping. The absorbance of light proved that FA_1-*x*_MA_*x*_PbI_3_ perovskites were boosted in the visible region and are in good agreement with recently published works. Furthermore, with a molar ratio of 50%, the bandgap energy for the mixed-structured perovskite (FA_0.5_MA_0.5_PbI_3_) was reduced to 1.472 eV, compared to 2.288 and 1.547 eV for $\delta$-FAPbI_3_ and MAPbI_3_, respectively. Numerical simulation using SCAPS-1D software was proposed to predict the effect of hybrid organic-cation perovskites on the performance of solar cell devices. The simulation model was successfully verified by comparing it with FA-based solar cells performance parameters reported in recent literature. The variation of absorber layer thickness on the device performance was also investigated, indicating that an optimal thickness range exists from 0.8 to 2.0 µm for preparing efficient solar cells. It was evidenced that the proposed planar n–i–p perovskite device (FTO/IL_1_/TiO_2_/IL_2_/FA_0.5_MA_0.5_PbI_3_/IL_3_/Spiro-OMeTAD/Au) shows a better performance with an efficiency of 26.22% compared to 8.43 eV for FAPbI_3_-based solar cell.

## Data Availability

All data generated or analyzed in this study are available from the corresponding authors upon any reasonable request.
